# HAM-TBS: high-accuracy methylation measurements via targeted bisulfite sequencing

**DOI:** 10.1186/s13072-018-0209-x

**Published:** 2018-07-04

**Authors:** Simone Roeh, Tobias Wiechmann, Susann Sauer, Maik Ködel, Elisabeth B. Binder, Nadine Provençal

**Affiliations:** 1 Department of Translational Research in Psychiatry, Max Planck Institute of Psychiatry, Kraepelinstr. 2-10, 80804 Munich, Germany; 2 Department of Psychiatry and Behavioral Sciences, Emory University Medical School, 12 Executive Park Dr NE #200, Atlanta, GA 30329 USA; 3 Faculty of Health Sciences, Simon Fraser University, 8888 University Drive, Burnaby, BC V5A 1S6 Canada; 4 Healthy Starts Theme, BC Children’s Hospital Research Institute, 938 West 28th Avenue, Vancouver, BC V5Z 4H4 Canada

**Keywords:** Targeted bisulfite sequencing, DNA methylation, Next-generation sequencing, 5-methylcytosine, *FKBP5*

## Abstract

**Background:**

The ability to accurately and efficiently measure DNA methylation is critical to advance the understanding of this epigenetic mechanism and its contribution to common diseases. Here, we present a highly accurate method to measure methylation using bisulfite sequencing (termed HAM-TBS). This novel method is able to assess DNA methylation in multiple samples with high accuracy in a cost-effective manner. We developed this assay for the *FKBP5* locus, an important gene in the regulation of the stress system and previously linked to stress-related disorders, but the method is applicable to any locus of interest.

**Results:**

HAM-TBS enables multiplexed analyses of up to 96 samples and regions spanning 10 kb using the Illumina MiSeq. It incorporates a triplicate bisulfite conversion step, pooled target enrichment via PCR, PCR-free library preparation and a minimum coverage of 1000×. TBS was able to resolve DNA methylation levels with a mean accuracy of 0.72%. Using this method, we designed and validated a targeted panel to specifically assess regulatory regions within the *FKBP5* locus that are not covered in commercially available DNA methylation arrays.

**Conclusions:**

HAM-TBS represents a highly accurate, medium-throughput sequencing approach for robust detection of DNA methylation changes in specific target regions.

**Electronic supplementary material:**

The online version of this article (10.1186/s13072-018-0209-x) contains supplementary material, which is available to authorized users.

## Background

DNA methylation is the covalent addition of a methyl group at the 5-carbon ring of cytosine, resulting in 5-methylcytosine (5mC). In the mammalian genome, this occurs predominantly in the context of CpG dinucleotides. It is one of several epigenetic marks influencing gene expression and serving multiple other purposes such as genomic imprinting, X chromosome inactivation and maintenance of genomic stability [1, 2]. Aberrant regulation of the establishment, maintenance, erasure or recognition of DNA methylation has been associated with a range of disease phenotypes [3, 4]. In addition, lasting effects of environmental risk factors may be reflected by changes in DNA methylation [5]. The need to measure DNA methylation in large human cohorts in a cost-effective manner is therefore of increasing interest for research in epidemiology and medicine [6].

**Fig. 1:**
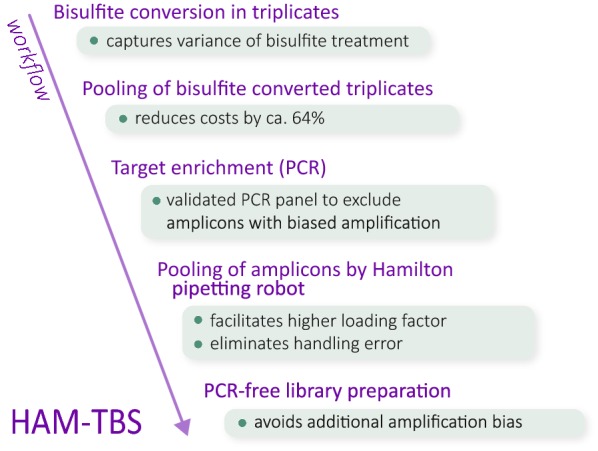
Workflow of the HAM-TBS method, depicting important processing steps and their advantages

Assessing DNA modifications with high accuracy and sensitivity in candidate loci would increase the power to detect and replicate such effects as well as to perform time course experiments in large numbers of samples to understand the stability of the environmentally induced changes during development. In addition, changes related to specific environmental exposure may only be present in specific cell types, although most studies rely on more complex tissues such as postmortem brain or blood samples. Assessing these effects in mixed tissues requires high accuracy in order to detect small changes emerging from a small number of cells. DNA bisulfite treatment followed by next-generation sequencing enabled the quantification of DNA methylation marks at single-base resolution. However, genome-wide bisulfite sequencing, although the best approach to identify DNA modifications, is still too cost intensive to be applied to large human cohorts at the coverage needed (> 60×) to detect differentially methylated sites [6]. Another set of accurate and cost-efficient measurement methods for DNA methylation at single CpG level are Illumina DNA methylation arrays. However, the ones currently available lack coverage in key enhancer regions that are important for environmentally driven changes and have a relatively small number of probes (~ 10–13) covering each site. Targeted bisulfite sequencing (TBS) offers a candidate approach to perform such studies with high resolution by increasing depth of read coverage per CpG to detect small changes in DNA methylation in a cost-efficient manner. Recently, few applications of TBS have been developed with differences in accuracy, throughput and library preparation [7–10]. Our TBS approach focuses on the *FKBP5* gene, which encodes the FK506-binding protein (FKBP51), a co-chaperone tightly involved in stress regulation. Genetic and epigenetic factors have repeatedly been shown to increase the activity of this gene and associated with increased stress-reactivity and psychiatric disorders [11]. We have previously reported allele-specific demethylation of CpG sites located in intronic enhancer regions of *FKBP5* specific to posttraumatic stress disorder (PTSD) in patients who had experienced child abuse [12]. These gene × environment interactions (GxE) may be mediated by differential susceptibility to adversity-induced changes in DNA methylation in specific enhancers. Current methods do not cover the relevant enhancer regions of *FKBP5* affected by stress exposure. A highly accurate, cost- and time-efficient method to investigate *FKBP5* DNA methylation in a large number of samples is thus critical to gain more insight into how DNA methylation changes may mediate these GxE. In this manuscript, we present a cost-effective, high-accuracy methylation measurement TBS (HAM-TBS) method to assess the regulatory regions of the FKBP5 locus. Incorporating a triplicate bisulfite conversion step, PCR-free library preparation and rigorous quality control (validation of PCR target sites, > 95% bisulfite conversion efficiency and 1000× coverage minimum) ensures that our method is extremely robust (Fig. 1). Medium throughput and handling accuracy of up to 96 samples spanning approximately 10 kb is facilitated by embedding the Hamilton pipetting robot and TapeStation with the Illumina MiSeq sequencer.

## Results

### QC, validation and optimization of the HAM-TBS method

**Fig. 2:**
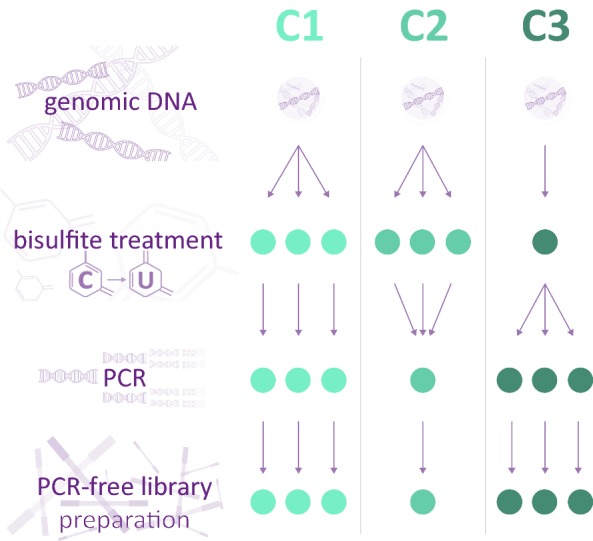
Setup of the TBS validation approach with the control conditions C1, C2 and C3. C1 is the reference condition with replicates in the bisulfite treatment and target enrichment step. C2 and C3 are more cost-effective versions dropping the replicate bisulfite treatment or target enrichment, respectively

**Fig. 3:**
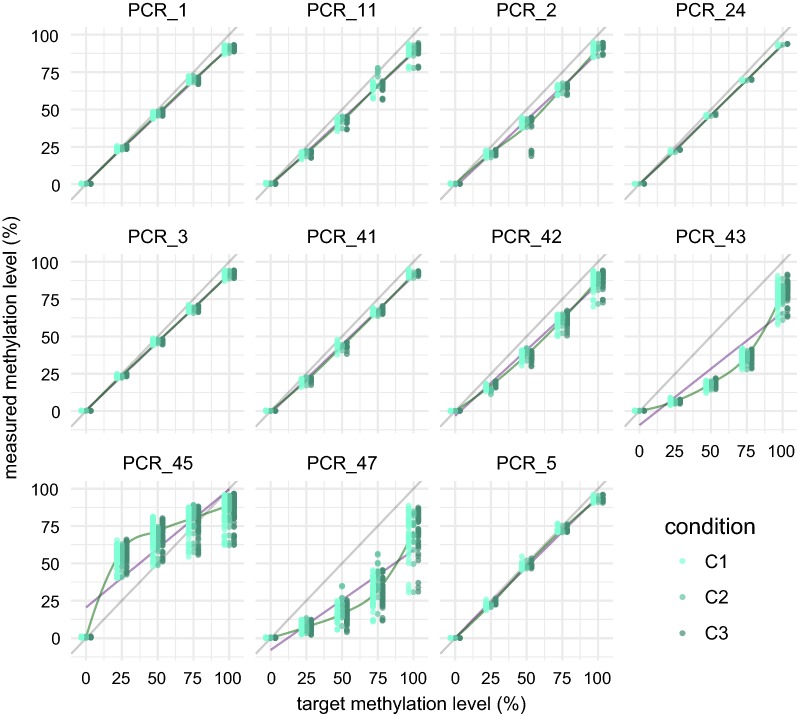
Methylation quantification of the control DNA used to evaluate the technical variability. Linear regression line (purple), Loess fit line (green). PCR_3 was excluded due to low coverage, PCR_47 was excluded due to low coverage and nonlinear amplification, and PCR_43 and PCR_45 were excluded due to nonlinear amplification

TBS is based on bisulfite conversion coupled with targeted enrichment via PCR, library preparation for sequencing and subsequent quantification of methylation levels. All steps are necessary and may influence the outcome by introducing bias to the assessment of methylation levels or by insufficient quality control of the data. The standard approach to minimize potential biases before sequencing is to produce replicates and assess the mean methylation levels during the analysis. In order to design a highly accurate yet cost-effective approach that is amenable to multiplexing, we assess at which step (bisulfite conversion or amplification) and to what extent technical variability would be introduced, as well as which quality control steps need to be performed on the sequencing data to ensure a robust analysis. To this end, we assessed the methylation level of 0, 25, 50, 75, 100% in vitro methylated bacterial artificial chromosome (BAC) control DNA for 3 different combinations of pooling strategies during the bisulfite treatment and PCR amplification (Fig. 2). Condition 1 (C1) assessed the methylation levels of control DNA using triplicate bisulfite treatments and PCR amplification for each replicate. C1 was considered the standard reference condition since each step was performed in triplicates. In condition 2 (C2), triplicate bisulfite treatments were pooled to perform one PCR amplification reducing the costs by approximately 64%. Finally, in condition 3 (C3) one bisulfite treatment of the control DNAs was performed followed by 3 separate PCR amplifications to assess the extent of the target enrichment bias. A smaller panel of 11 different PCRs (Fig. 3) within the *FKBP5* locus (see table in Additional file 1) served as basis for this analysis. Before comparing the three conditions, the collected sequencing data were subjected to three quality control steps in order to ensure accurate assessment of minimal methylation levels as well as small changes between samples.

**Fig. 4:**
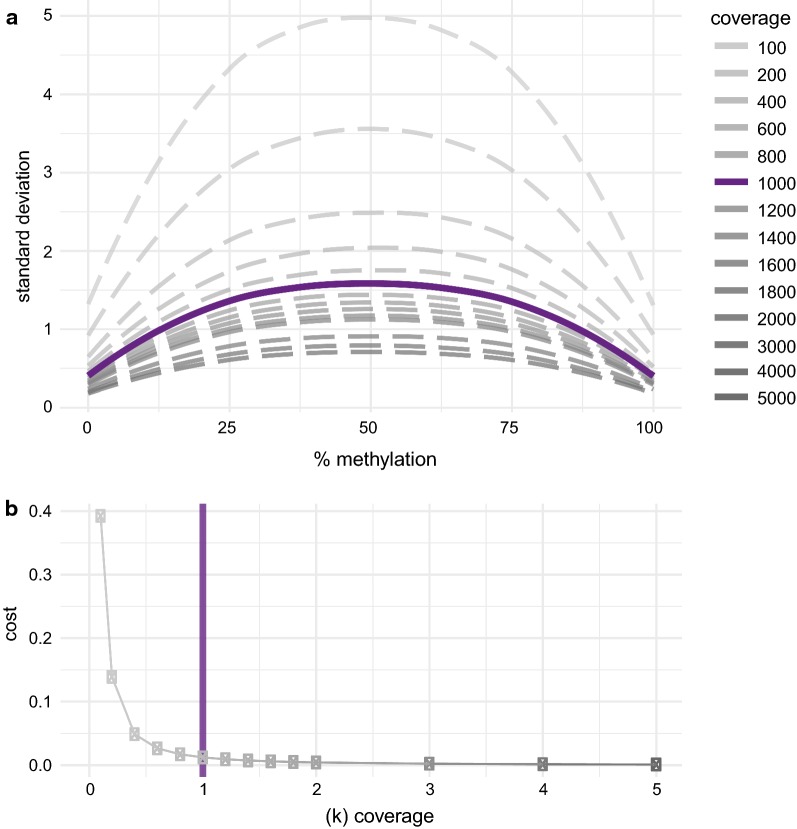
**a** Standard deviation of varying coverage with respect to methylation level. **b** Cost (accuracy as sum of the standard deviation) with respect to increasing coverage

1. *Bisulfite conversion rate *> *95%*. We assessed the bisulfite conversion rate per sample and per amplicon and excluded rates lower than 95% from the analysis.
2. *Removal of PCR artefacts* During the target amplification, the PCR occasionally introduces artefacts presenting non-existent CpG sequences in the target region. They present at very low coverage and extreme levels of methylation (~ 0 or ~ 100%). In order to not exclude potential SNPs giving rise to CpGs, we removed artefacts on this basis rather than limiting the analysis to known CpGs according to the reference genome.
3. *Minimum coverage of 1000 *×Higher sequencing depth and coverage of the CpGs yields higher accuracy of the methylation quantification. In order to determine the right balance between sequencing depth and thereby cost and sufficient accuracy, we took random subsamples of varying sequencing depth of an in silico created library representing methylation levels from 0 to 100% and assessed the standard deviation for each level of methylation with respect to coverage (Fig. 4a). To find a meaningful cutoff for coverage, we considered the trade-off between sum of the average standard deviation per amplicon (cost) present in various levels of coverage (Fig. 4b). In accordance with previous findings [7], we identified 1000× coverage as a useful cutoff for our analysis, as the gain in accuracy with increasing coverage above this threshold is low and 1000× is reasonable to achieve for a larger locus, e.g., 9 kb in the *FKBP5* panel.

**Fig. 5:**
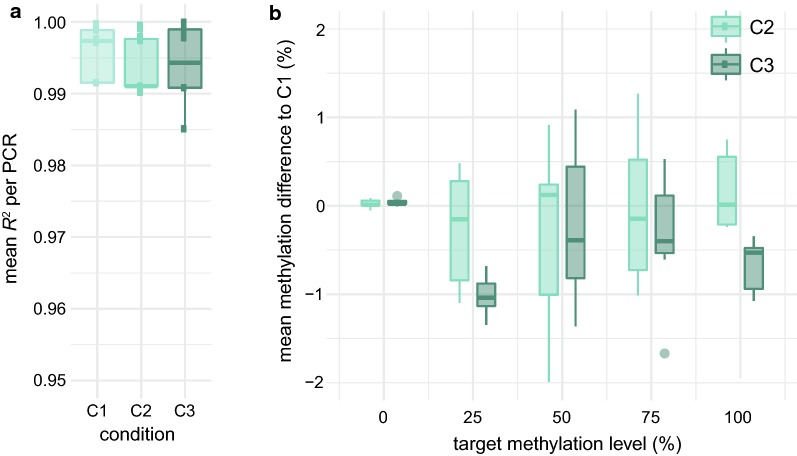
**a** Mean *R*^*2*^ per amplicon for all conditions C1, C2 and C3. **b** Mean methylation difference per PCR for C2 and C3 to the clean control condition C1. *x*-axis: target methylation levels during in vitro methylation

All PCRs for our validation experiment showed bisulfite conversion levels > 99%. After QC, a total of 40 CpG spread across 7 amplicons remained in our analysis (1 PCR failed due to coverage < 1000×, 1 showed nonlinear amplification and coverage < 1000×, 2 showed nonlinear amplification). Methylation levels were very similar between all 3 conditions with an average error of < 1% when comparing absolute methylation levels of C2 and C3 versus C1 (Fig. 5b). We calculated the *R*^*2*^ values for each assessed CpG across the titration levels and used the mean per amplicon to compare the 3 conditions. *R*^*2*^ is a measure for assessing linearity of amplification of the methylation signal, which is crucial when quantifying methylation changes in, e.g., cohort studies. Again, all conditions showed very high mean *R*^*2*^ values above 0.99 (Fig. 5a). This confirms that all conditions are suitable for high-accuracy methylation detection. The introduced biases in our workflow, based on the control DNA, are minimal and enable very accurate methylation quantification even without including triplicates for the bisulfite conversion or target amplification. However, opposed to the target amplification, we cannot exclude slightly elevated variance of the bisulfite conversion on non-in vitro methylated DNA from, e.g., patients. Therefore, we chose to use C2 for our HAM-TBS method. While it still maintains a triplicate bisulfite conversion step, it is the most cost-effective of the tested conditions, an important factor when processing many samples from cohort studies.

### Comparison of the technical accuracy of pyrosequencing to TBS

Next, we aimed to compare TBS to pyrosequencing, the reference method used for targeted DNA methylation analysis. We assessed the methylation levels of 5 CpGs within PCR_5 and PCR_11 measured by pyrosequencing as well as using HAM-TBS with the C1 protocol. The methylation analysis using pyrosequencing showed a high mean standard deviation of 4.68% with a maximum SD of 14.56%. The analysis using next-generation sequencing with C1 showed a much lower mean standard deviation of 0.72% with a maximum SD of 1.83%. This demonstrates a significantly lower technical variation and therefore higher accuracy when assessing methylation levels using a TBS approach.

### Development of an extensive HAM-TBS *FKBP5* panel covering relevant regulatory sites

**Fig. 6:**
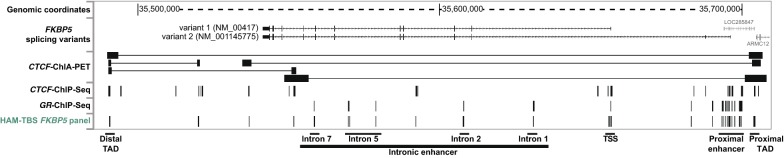
Genome browser shot (hg19) illustrating the HAM-TBS *FKBP5* panel and important locus-specific data. ***CTCF*-ChIA-PET**: track indicating the locations of *CTCF* factor-mediated chromatin interactions determined by Chromatin Interaction Analysis with Paired-End Tag (ChIA-PET) data (GM12878 [24]). ***CTCF*** **ChIP-seq** and ***GR*** **Chip-seq**: regions of transcription factor binding derived from chromatin immunoprecipitation (ChIP) experiments in multiple cell lines from the ENCODE project; **HAM-TBS** ***FKBP5*** **panel**: locations of the amplicons contained in this panel

*FKBP5* is an important gene in the field of psychiatry. The gene is larger than 100 kb rendering the assessment of the full locus including the adjacent up- and downstream regions unfeasible and too cost intensive for TBS methods. We thus restricted our analysis to functionally relevant sites of interest to ensure compatibility with targeted measurement methods and enable the assessment in large cohorts. To this end, we designed and validated a comprehensive amplicon panel (Fig. 6) including the TSS, TAD boundaries, intergenic and proximal enhancers as well as *GR* and *CTCF* binding sites (see methods for further details). The resulting HAM-TBS *FKBP5* panel is composed of 29 amplicons passing our QC’s threshold (described above) and covering 315 CpGs across the locus. The sequencing data showed sufficient bisulfite conversion for all amplicons when performed on control DNA using C2. In total, 27 of the amplicons included in the panel presented good linearity (see figure in Additional file 2) across the assessed methylation levels. Two amplicons located near the TSS showed a mild PCR bias, where methylation levels were lower than expected for the 50% and 75% controls (PCR_7, PCR_9). These amplicons have a very high CpG content of > 25%; hence, CpGs in the primer could not be avoided. It has been previously shown that methylation levels in this region are very low (< 5%) across tissues [12], so that any bias at higher methylation levels would not impair accurate quantification of this region. We thus incorporated sites located in this region in the panel, but they should be used with caution if higher methylation levels are observed. PCR_26 of the HAM-TBS *FKBP5* panel is located in the *H19* locus [13] which is an imprinted gene and serves as an internal positive control with an expected methylation level ~ 50%.

### Application and costs

The HAM-TBS method can be multiplexed up to 96 samples in a medium-throughput manner. To demonstrate the applicability of our approach, quality control statistics of data derived from an experiment containing 95 blood samples from patients and the full *FKBP5* panel of 29 amplicons are described here. After reads mapping and methylation calling, we identified PCR artefacts comprising ~ 1% of the methylation sites and removed them from the data, and 9 samples in 1 PCR showed insufficient bisulfite conversion rates (< 95%) and were also removed. Two loci were identified as SNPs giving rise to a CpG sites in patients. In total, 91% of sample x amplicon data passed our filtering criteria. 27 amplicons passed QC with sufficient coverage and quality in > 75% of samples, while two amplicons were dropped due to < 1000× coverage (Additional file 3A, B). The control amplicon spanning the H19 imprinted locus for which methylation level is known to be ~ 50% [14] shows the expected methylation profile in all samples (Additional file 3C). HAM-TBS approach allowed the quantification of 276 methylation sites for 95 samples in one single MiSeq run.

An assessment of the relative costs for each of the main reagents for this experiment containing 96 samples (95 patients and unmethylated control) with increasing number of amplicons assessed is depicted in Additional file 4. The quantifications using TapeStation and the PCR-free library preparation are the two most cost-intensive steps. The proportion of costs for the amplicon quantification using the TapeStation increases with the higher amount of amplicons investigated, while relative costs for the library preparation and sequencing chemistry decrease with the inclusion of more amplicons.

## Discussion

We developed a targeted medium-throughput approach for measuring DNA methylation levels in multiple samples in parallel. This method enables cost-efficient high-resolution methylation measurements of target loci in cohorts of patients and probands at the *FKBP5* gene, a locus with large interest in the psychiatric and psychological community [11]. This cost-efficient, accurate method to determine *FKBP5* methylation levels would thus serve a large number of researchers. Our method is positioned between whole genome bisulfite sequencing and targeted approaches as pyrosequencing. The first is expensive and yields lower coverage and accuracy of single CpGs; the latter only allows to assess very small regions at a time and can generate significant variance between replicates. HAM-TBS enables the analysis of a targeted but larger region (~ 10 kb) at high resolution and low costs. DNA methylation studies in large cohorts, investigating the impact of environment or association with disease status in mixed tissues, necessitate high accuracy at single-site resolution. In fact, TBS was able to resolve methylation levels with a mean accuracy of 0.72%. A high level of accuracy was maintained in more cost-efficient approaches using only one PCR amplification round. By pooling triplicate bisulfite treatments prior to PCR amplification, we can account for variance introduced by the bisulfite treatment but also reduce costs and hands-on time during the target amplification.

The accuracy of the method benefits from a PCR-free library preparation and rigorous quality control (prior evaluation of linear PCR amplification of the target site, bisulfite conversion efficiency > 95% and read coverage minimum of 1000×). Nonetheless, a proper assessment of possible amplification biases due to the choice of amplicon location in the design step is critical. Some loci can show nonlinear amplification curves, which renders them inappropriate for methylation quantification. Adjustment of primer design and PCR conditions may help solve this issue, but for some loci optimization may not be possible. For instance, in CpG islands with high CpG density, we observed that amplification curves were not linear, revealing a bias which became more pronounced as the level of methylation increased. Differential methylation results from these sites should be interpreted with caution and perhaps require additional replication. Besides validating each amplicon prior to usage, including controls such as in vitro unmethylated DNA, water and endogenous hemimethylated region, the H19 locus, during each HAM-TBS experiment is important and enables quality checks for each step of the protocol.

Additionally, reaching 1000× coverage is an important step to provide high resolution on methylation changes [8]. However, accurate quantification and pooling of many amplicons across multiple samples while reaching sufficient coverage of all regions has limitations. In theory, even though the MiSeq can handle a much higher loading factor (amplicons x samples) of almost 20,000 (disregarding uneven pooling of libraries, filtering of reads due to low quality or high amounts of PhiX), a maximum of 2500–3000 has proven to be feasible with minimal dropout rates. Assuming multiplexing of 96 samples and 25 amplicons at an average length of 400 bp, a region of approximately 10 kb can be comfortably covered with this approach. Notably, we streamlined the method to handle loading factors > 2000 by implementation of Agilent’s TapeStation and a pipetting robot for quantification and pooling of amplicons. Besides the throughput, this improves the robustness of the workflow. Our approach is designed to match the specifications of the Illumina MiSeq with its ability to run for 600 cycles resulting in 300 bp-long paired-end reads. This enables full-length coverage of amplicons up to a length of 600 bp. While our approach can be applied to different sequencers, such as the Illumina HiSeq for example, it would be necessary to design shorter amplicons due to the current limits of the sequencing chemistry. Using another sequencer, it is important to mention the index hopping phenomenon on the Illumina platforms [15]. It is less present on the MiSeq compared to other machines with pattern flow cells as our data show consistent levels of methylation close to 0% across all in vitro unmethylated control samples indicating no issue with this specific bias. Nonetheless, it should be kept in mind that approaches like unique dual indexes when available or Illumina’s Free Adapter Blocking Reagent are recommendable and gain importance, especially when using a different Illumina sequencer.

In the past years, only few TBS methods have been developed [8–10] with different methodological foci. Thus far, Bernstein et al. [10] allows a panel of 48 indices, while the approach by Chen et al. [9] could allow for a multiplexing rate of 1536 samples due to custom-made barcodes, but in practice only 478 have been used to date. In the latter method, the high multiplexing capacity comes at the cost of an additional PCR step potentially introducing additional bias. Moreover, increasing the number of samples needs to be weighed against the size of the target region in order to ensure sufficient coverage. We identified 1000× coverage as an optimal cutoff in terms of accuracy and cost in agreement with a publication by Masser et al. [8]. In the above-described study by Chen et al. [9], 100× was used as minimum cutoff. Based on our in silico analysis (Fig. 4a), this would lead to less accurate quantification of methylation levels. Besides the number of samples that can be processed, the size of the region of interest is also an important factor to be considered. The method by Masser et al. [8] has been applied to 2 amplicons (233 and 320 bp), while Chen et al. enable the assessment of larger loci around 10 kb—comparable to our HAM-TBS approach. Lastly, amplification-based library preparation methods have been adapted by most TBS approaches. At this point, HAM-TBS utilizes a PCR-free library preparation to avoid adding amplification biases.

Finally, using the optimized HAM-TBS workflow, we designed a panel comprising 29 amplicons to accurately assess methylation within the *FKBP5* locus using HAM-TBS. This panel covers ~ 9 kb and targets important regulatory regions of the *FKBP5* gene including the TSS, intergenic and proximal enhancers and TAD boundaries including *CTCF* binding sites. The HAM-TBS method and the *FKBP5* panel present valuable tools for epigenetic studies in which a highly accurate assessment of methylation levels is critical such as GxE studies in psychiatric research. It allows cost-efficient quantification of methylation in larger cohorts with optimized hands-on time due to automatization.

## Conclusion

The presented method HAM-TBS offers a robust and low-cost method for researchers interested in DNA methylation measurements of specific target regions. In addition, we supply a validated panel of 29 amplicons to assess methylation levels of important regulatory regions in the *FKBP5* locus, a gene of great interest in the field of psychiatry.

## Methods

### Generation of in vitro methylated control DNA

All primers designed for bisulfite PCR were first tested on in vitro methylated DNA to assess amplification efficiency and bias. For PCRs within the *FKBP5* gene, an in vitro methylated BAC (RP11-282I23, BACPAC) was used to generate control DNA. For PCRs outside the *FKBP5* locus (PCR_26, PCR_34, PCR_35), genomic DNA extracted from whole blood was amplified using the REPLI-g Mini Kit (QIAGEN GmbH, Hilden, Germany) to generate unmethylated DNA. 100% methylated DNA was achieved using in vitro methylation with M.SssI methyltransferase. After a first incubation (3 µg DNA, 0.5 µl SAM (32 mM), 1 µl M.SssI (20 U/µl, 40 µl NEB buffer 2 [10×], diluted with ddH2O up to 400 µl) of 4 h at 37C, 1 µl of M.SssI (20 U/µl) and 1 µl of SAM (32 mM) were added, and a second 4-h incubation was performed. Subsequently, the reaction was purified using the nucleotide removal kit (QIAGEN GmbH, Hilden, Germany). In vitro methylation was repeated with the eluted DNA for a second time. 25, 50 and 75% methylated control DNA was obtained by mixing 0 and 100% DNAs. In vitro methylation of control DNA was checked via pyrosequencing.

### Bisulfite treatment of DNA

We used the EZ DNA Methylation Kit (Zymo Research, Irvine, CA) in column and plate format depending on the amount of DNA and throughput needed. Between 200 and 500 ng was used as input DNA and processed according to the manufacturer’s instructions. DNA was eluted twice in 10 µl elution buffer which recovered over 90% of the input DNA after bisulfite conversion when using the column format. In order to quantify bisulfite treated DNA, we use a spectrophotometer with RNA quantification settings.

### Target enrichment and amplicon pooling

The amplification of target locations from converted DNA (20 ng per amplicon) was achieved using the TaKaRa EpiTaq HS Polymerase (Clontech, Mountain View, CA; final concentration: 0.025 U/l), bisulfite-specific primers (final concentration of each primer: 0.4 M) and a touchdown cycling protocol with 49 cycles [for more details (see table in Additional file 5 and section HAM-TBS *FKBP5* panel). The amplicons of all PCR reactions were quantified using the Agilent 2200 TapeStation (Agilent Technologies, Waldbronn, Germany] and equimolar pooled with the Hamilton pipetting robot. After speed-vacuum and resuspension in 50 µl, a double-size selection was applied using Agencourt AMPure XP beads (Beckman Coulter GmbH, Krefeld, Germany) to remove excess of primers and genomic DNA.

### Control samples

For every TBS run, we included three different controls. First, up to three water controls in order to monitor cross-contamination with DNA and detect if the plate was accidentally rotated. Second, an unmethylated control DNA as a positive control and to detect failed steps throughout the workflow. And third, the H19 locus which is an imprinted region and presents with methylation levels ~ 50% as a positive control for bisulfite conversion in genomic DNA and detect outliers in patient samples. An amplicon located at this locus is incorporated in the *FKBP5* panel.

### Library preparation and sequencing

For library generation, Illumina TruSeq DNA PCR-Free HT Library Prep Kit (Illumina, San Diego, CA) was used according to the manufacturer’s standard protocol and obtained high-quality libraries using 500 ng of starting material (during optimization, input amounts as low as 100 ng were tested and showed no loss of quality on the QC level). Qubit 1.0 (Thermo Fisher Scientific Inc., Schwerte, Germany) was used for quantification, Agilent’s 2100 Bioanalyzer (Agilent Technologies, Waldbronn, Germany) for quality assessment and Kapa HIFI Library quantification kit (Kapa Biosystems Inc., Wilmington, MA) for final quantification before pooling. Libraries were pooled equimolarly. Sequencing of the libraries was performed on an Illumina MiSeq using Reagent Kit v3 (Illumina, San Diego, CA; 600 cycles) in paired-end mode, with 30% PhiX added.

### Sequencing data processing

First, read quality was verified using FastQC [16]. Adapter sequences were trimmed using cutadapt v.1.9.1 [17]. For alignment to a restricted reference of hg19 based on the PCR locations, Bismark v.0.15.0 [18] was used. Due to the 600-cycle sequencing chemistry, PCRs shorter than 600 bp produce overlapping paired-end reads. Using an in-house developed Perl script, we trimmed low-quality overlapping ends. Quantification of methylation levels in CpG and CHH context was performed using the R package methylKit [19] with a minimum quality score of 20. The methylation calls were subjected to 3 quality control steps. First, we considered CHH levels for each sample and excluded samples if the conversion was less than 95% efficient. Second, we filtered PCR artefacts introduced by PCR amplification errors giving rise to CpG sites in some reads. As we do not restrict the analysis to known CpG sites, every read indicating the presence of a CpG will be considered and the information extracted. These artefacts mostly present at very low levels of coverage and 0 or 100% methylation. Lastly, according to our coverage cutoff, we excluded CpG sites supported by less than 1000 reads. Subsequent analysis comparing methylation levels from the conditions C1, C2 and C3 as well as data from pyrosequencing was performed in R.

### Coverage considerations

When performing a sequencing experiment, one will usually sequence part of the generated library and quantify the methylation levels on this basis rather than sequence the whole library to see the true level within. Therefore, each sequencing experiment corresponds to drawing a random subset of a certain size (sequencing depth) of the whole library and can be viewed as a subsampling problem. Depending on the sequencing depth, this will yield a different level of accuracy of the methylation levels. We created a dataset simulating CpGs methylated at levels from 0 to 100% supported by 100,000 “fragments” each. Therefore, e.g., for 10% methylation level, a set 10,000 Cs and 90,000 Ts was created. Accordingly, sets for 0–100% methylation were created. Using a bootstrapping approach, we drew 1000 random subsets of varying sequencing coverage (100, 200, 400, …, 2000, 3000, 4000, 5000) from each set representing a certain level of methylation and the standard deviation (SD) was calculated. As a proxy for the increase in accuracy versus increase in sequencing depth (costs), the combined SD was divided by the sequencing depth. Of note, this is in concordance with results from the same analysis on highly covered amplicon data from our laboratory (data not shown).

### Pyrosequencing

Methylation analysis by pyrosequencing of 5 CpGs covered within PCR_5 (CpG 35607969, CpG 35608022) and PCR_11 (CpG 35690280, CpG 35690318, CpG 35690365) was performed in triplicates on BAC control DNA. Bisulfite conversion of in vitro methylated control DNA was applied as described above. Target enrichment by PCR was achieved with a biotinylated reverse primer but otherwise performed as described above. Pre-treatment of PCR amplicons was facilitated with the PyroMark Q96 Vacuum Workstation (QIAGEN GmbH, Hilden, Germany). Sequencing of *FKBP5* CpGs was performed on a PyroMark Q96 ID system using PyroMark Gold Q96 reagents (QIAGEN GmbH, Hilden, Germany) and sequencing primers according to Klengel et al. [12]: P4 S1 (TTTGGAGTAGTAGGTTAAA) GRE3 S1 MPI (GGGAATTATGAGGTTG). The PyroMark Q96 ID Software 2.5 (QIAGEN GmbH, Hilden, Germany) was used for data analyses.

### HAM-TBS *FKBP5* panel

We designed 29 primer pairs (see table in Additional file 5) using BiSearch [20, 21] targeting the *FKBP5* locus. Initially, 32 PCRs were included, but 3 PCRs were not selected for the panel due to QC failure. The excluded amplicons showed nonlinear amplification due to an elevated GC content in the region. Positions of amplicons covering glucocorticoid response elements (GREs) were selected from Klengel et al. [12] and the *GR* ChIP-Seq from the ENCODE project [22]. Amplicons covering *CTCF* binding sites were selected using HI-C peaks [23], *CTCF*-ChIA-Pet interactions from a lymphoblastoid cell line (GM12878, Tang et al. [24]) and *CTCF* ChIP-Seq information from the ENCODE project [22]. Lastly, amplicons located near the TSS were included in the panel. Only primers without CpGs in their sequence were chosen, with the exception of 2 amplicons close to the TSS where this could not be avoided due to the high CpG content of the region. The selected amplicons ranged from 200 to 450 bp in length.

## Additional files

**Additional file 1.** A table containing the genomic coordinates of a smaller panel of 11 amplicons located within the *FKBP5* locus. These amplicons were used to assess the source of potential biases as well as variability between replicates.

**Additional file 2.** A figure displaying the bias assessment for all amplicons comprising the *FKBP5* HAM-TBS panel.

**Additional file 3.** A figure displaying the QC statistics of a HAM-TBS experiment with 95 samples using the *FKBP5* panel and methylation levels of the H19 locus.

**Additional file 4.** A figure displaying the relative and absolute costs for a HAM-TBS experiment with 96 samples.

**Additional file 5.** A table containing the genomic coordinates of the *FKBP5* HAM-TBS panel including primer sequences and cycling conditions.
