# Supplementary figures and images for: HAM-TBS: high-accuracy methylation measurements via targeted bisulfite sequencing
Source: Epigenetics Chromatin. 2018 Jul 4;11:39. doi: 10.1186/s13072-018-0209-x (PMC6031184; doi:10.1186/s13072-018-0209-x)

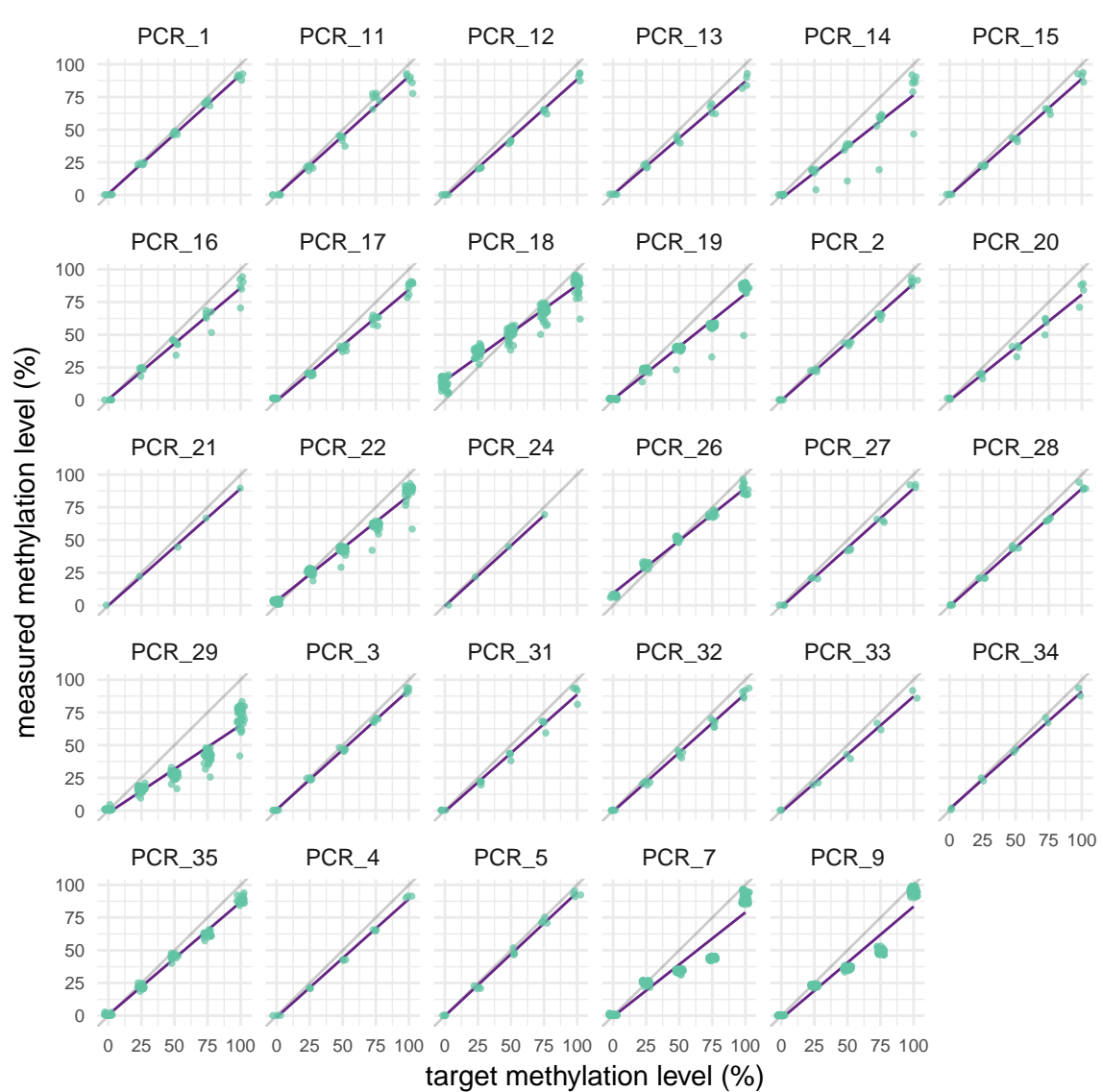

Supplement: Supplementary file 2 — Additional file 2. A figure displaying the bias assessment for all amplicons comprising the FKBP5 HAM-TBS panel. [file 13072_2018_209_MOESM2_ESM.pdf]

**A**

## Cost overview for 96 samples

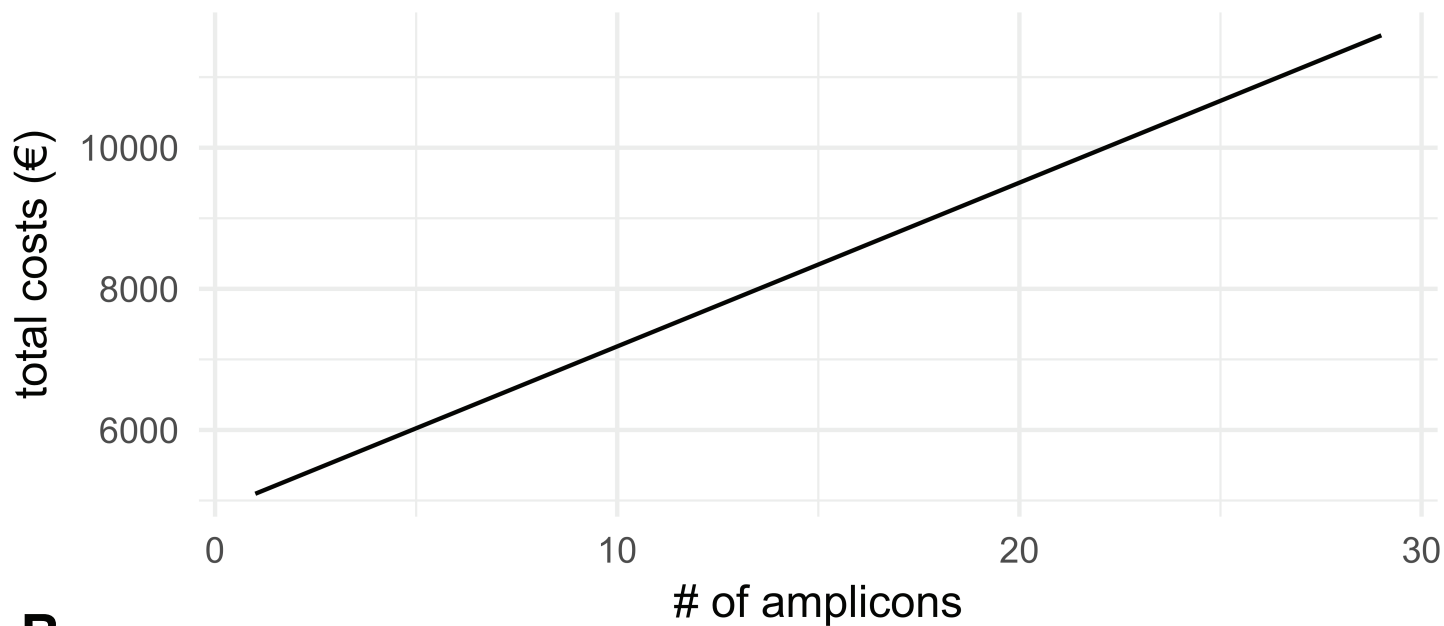**B**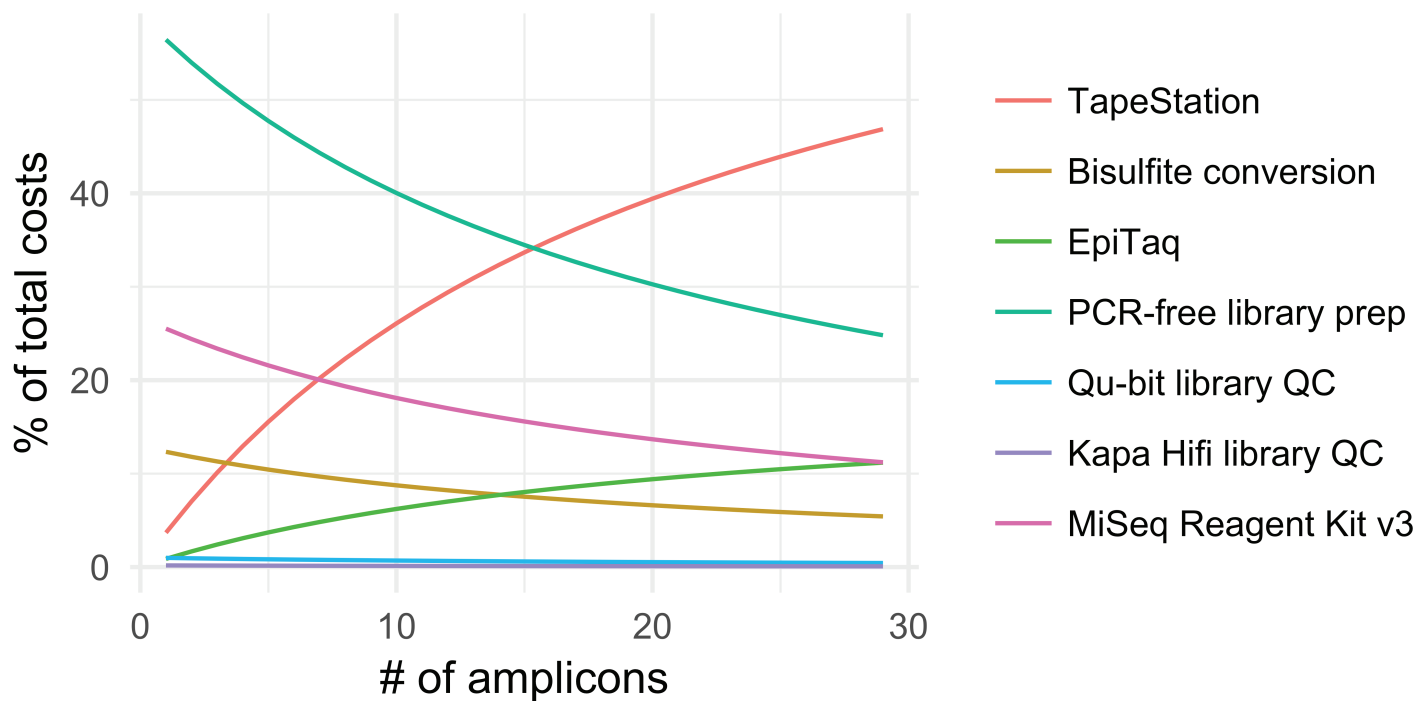

Supplement: Supplementary file 4 — Additional file 4. A figure displaying the relative and absolute costs for a HAM-TBS experiment with 96 samples. [file 13072_2018_209_MOESM4_ESM.pdf]
