# Supplementary material for: HAM-TBS: high-accuracy methylation measurements via targeted bisulfite sequencing
Source: Epigenetics Chromatin. 2018 Jul 4;11:39. doi: 10.1186/s13072-018-0209-x (PMC6031184; doi:10.1186/s13072-018-0209-x)
Supplement: Supplementary file 3 — Additional file 3. A figure displaying the QC statistics of a HAM-TBS experiment with 95 samples using the FKBP5 panel and methylation levels of the H19 locus. [file 13072_2018_209_MOESM3_ESM.pdf]

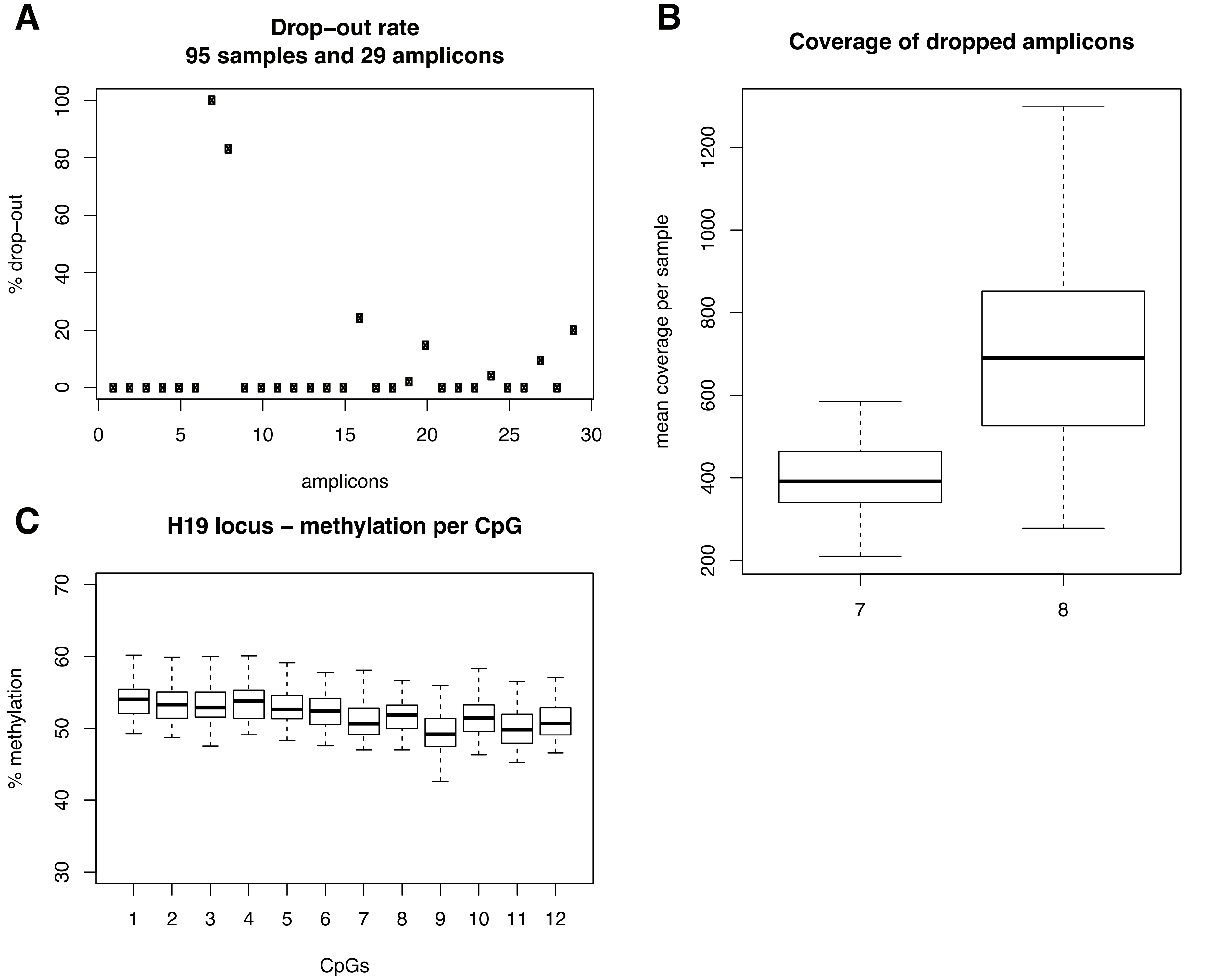
A
Drop–out rate
95 samples and 29 amplicons
% drop–out
amplicons
0
20
40
60
80
100
0
5
10
15
20
25
30
B
Coverage of dropped amplicons
mean coverage per sample
200
400
600
800
1000
1200
7
8
C
H19 locus – methylation per CpG
% methylation
CpGs
30
40
50
60
70
1
2
3
4
5
6
7
8
9
10
11
12
